# Trp101-Mediated Cold Adaptation in *Sphingomonas* sp. Thioredoxin: Increased α4-Helix Rigidity with Preserved Overall Flexibility

**DOI:** 10.1021/acsomega.5c07089

**Published:** 2025-11-04

**Authors:** Mohammed Shazaly A. Elhassan, Hoa Nguyen, ChangWoo Lee

**Affiliations:** † Department of Biomedical Science and Center for Bio-Nanomaterials, 37975 Daegu University, Gyeongsan 38453, South Korea

## Abstract

Thioredoxin (Trx) is a small, conserved redox protein composed of five β-strands and four α-helices, and it reduces disulfide bonds and helps maintain cellular redox homeostasis. During evolution from the Trx of the last bacterial common ancestor (LBCA Trx) to *Escherichia coli* Trx (EcTrx), the α3 helix became more flexible, while the α4 helix gained rigidity. To investigate the role of α4-helix rigidity in cold adaptation, we analyzed *Sphingomonas* sp. Trx (SpTrx), a cold-adapted ortholog, focusing on α2−α4 salt bridges and α4−β5 hydrophobic interactions. We constructed single and double mutants, targeting Glu43, Glu47, and Trp101, and assessed structural stability using thermal shift assays, chemical denaturation, fluorescence spectroscopy, and circular dichroism. Catalytic activity was evaluated using insulin reduction and DTNB-based kinetic assays. Salt bridge mutations (E43A, E47A, and E43A/E47A) modestly decreased both stability and enzyme activity. In contrast, hydrophobic interface mutations (W101A and W101F) caused more substantial destabilization, with W101A inducing the most pronounced structural disruption. The E47A/W101F double mutant exhibited poor expression and the lowest stability and activity. In a comparative study with EcTrx, salt bridge mutations had a greater impact on thermal stability than in SpTrx. While F102A significantly reduced stability and increased flexibility, F102W (the SpTrx-equivalent substitution) increased both stability and rigidity. These findings demonstrate that in SpTrx, Trp101 enhances α4-helix rigidity through hydrophobic packing while preserving overall flexibility. This study highlights the evolutionary divergence of stabilization mechanisms in Trx orthologs and provides insight into how cold-adapted enzymes maintain activity at low temperatures.

## Graphical abstract

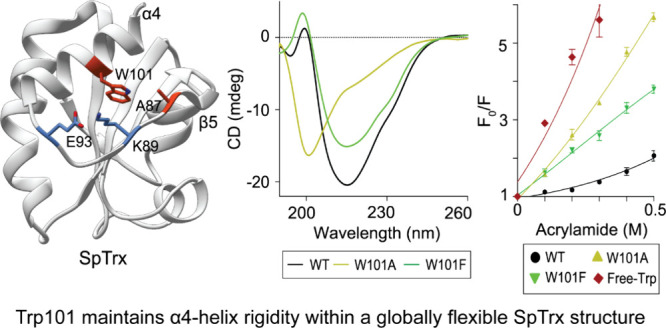

## 1 Introduction

Thioredoxins (Trxs) are small redox proteins (∼12 kDa) found in all living organisms.^1,2^ They catalyze thiol–disulfide exchange reactions via a conserved CXXC active-site motif, in which two Cys residues undergo reversible oxidation–reduction reaction.^3,4^ Trxs play essential roles in redox homeostasis, DNA synthesis, and signal transduction pathways by transferring electrons from β-nicotinamide adenine dinucleotide phosphate (NADPH) through Trx reductase to the reduced form of Trx.^5–7^ Trxs adopt a highly conserved Trx fold, consisting of a five-stranded central β-sheet surrounded by four α-helices (Figure 1A).^8–10^ This fold has been preserved for nearly four billion years, with the β-sheet remaining rigid and conserved.^11,12^ A buried Phe in the β2 strand plays a critical role in structural stability through tight hydrophobic packing.^13–15^

**Figure 1:**
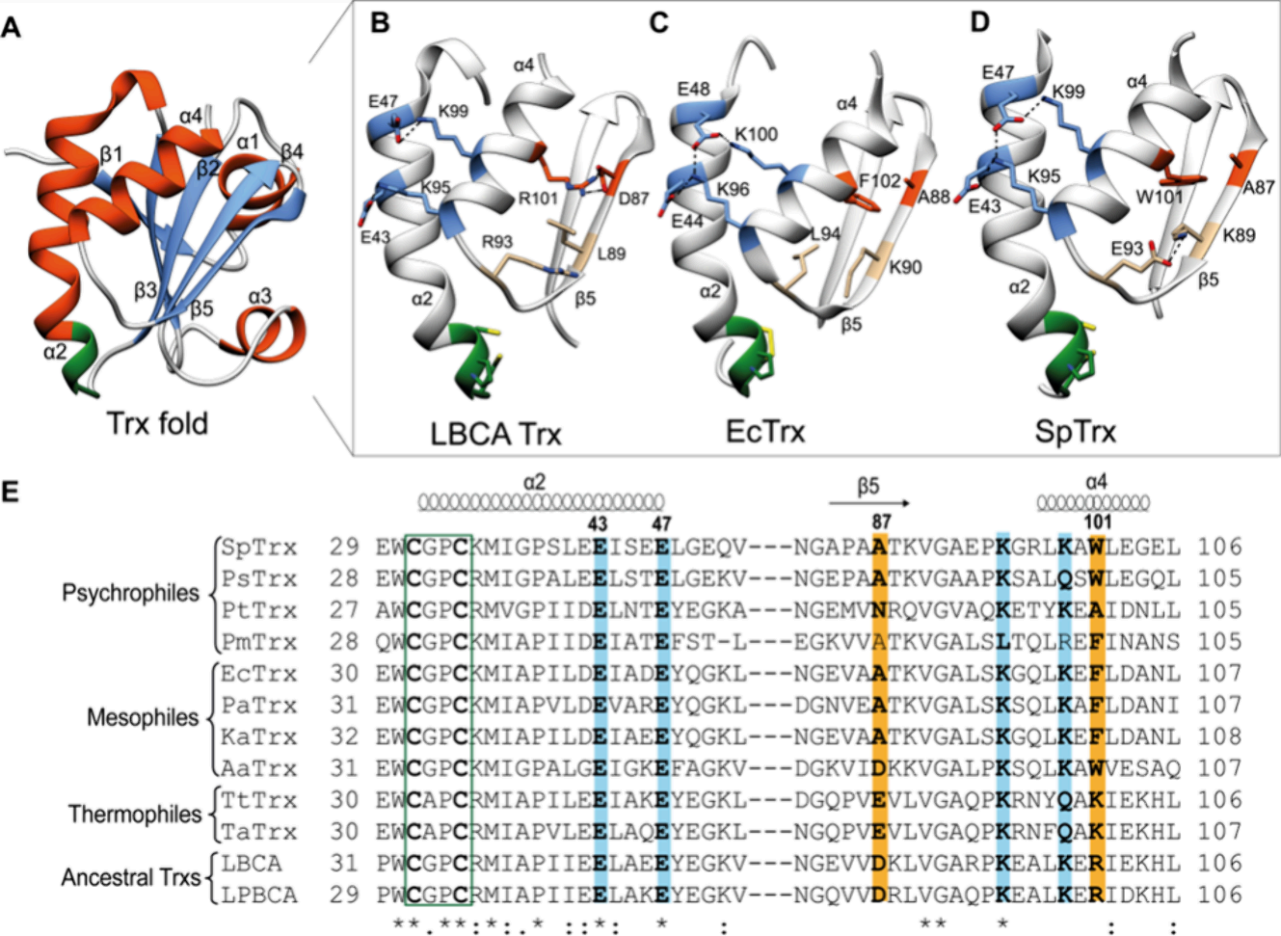
Structural and amino acid sequence comparison of Trxs. (A) Overall Trx fold showing the five-stranded central β-sheet surrounded by four α-helices, drawn using the EcTrx structure (PDB ID: 2TRX). (B–D) Structural comparison of the α2−α4−β5 region among the ancestral LBCA Trx, mesophilic EcTrx, and psychrophilic SpTrx. α2−α4 interactions (blue), α4−β5 interactions (orange), and the active-site motif (green) are indicated. (E) Multiple sequence alignment of Trxs adapted to different habitat temperatures. Psychrophilic Trxs: SpTrx (*Sphingomonas* sp. PAMC 26621, NCBI ID: WP_010164143) (AlphaFold 3-predicted model), PsTrx (*Pseudomonadota* bacterium, NCBI ID: MDQ3140117.1), PtTrx (*Psychroflexus torquis*, NCBI ID: WP_015023308.1); Mesophilic Trxs: EcTrx (*Escherichia coli*, PDB ID: 2TRX), PaTrx (*Pseudomonas aeruginosa*, NCBI ID: KJJ13934.1), KaTrx (*Klebsiella aerogenes*, NCBI ID: KLF75859.1), AaTrx (*Acetobacter aceti*, PDB ID: 2I4A); Thermophilic Trxs: TtTrx (*Thermus thermophilus*, PDB ID: 2CVK), TaTrx (*Thermus aquaticus*, NCBI ID: WP_053767495.1); Ancestral Trxs: LBCA (last bacterial common ancestor, PDB ID: 4BA7), LPBCA (last common ancestor of the Cyanobacterial, Deinococcus, and Thermus group, PDB ID: 2YJ7).

The Trx from LBCA exhibited enhanced thermal stability due to an increased number of salt bridges and optimized surface electrostatics.^12^ However, modern Trxs generally show reduced thermal stability compared to their ancestral counterparts.^16–18^ In EcTrx, structural adaptations include a longer α1−β2 loop and a larger aromatic pair between the α1 and α3 helices with reduced packing density, both contributing to increased flexibility.^19,20^ While the α3 helix has become more flexible, the α4 helix has become more rigid during evolution.^16,21^ Together with the neighboring β5 strand, the α4 helix has undergone additional modifications near the C-terminus. For instance, an ancestral α4−β5 salt bridge in LBCA Trx (Asp87–Arg101) has been replaced in EcTrx by a hydrophobic interaction (Ala88–Phe102), along with a stabilizing Leu89-to-Lys90 substitution that forms an internal hydrogen bond to a Gly backbone carbonyl, and an Arg93-to-Leu94 substitution that introduces a hydrophobic contact and enhances stability (Figure 1B,C).^12,22,23^ The destabilizing effects of mutations such as Phe102 to Ala/Arg and Leu103 to Ala in EcTrx further support the importance of hydrophobic contact at the α4−β5 interface in maintaining Trx stability.^22,24^

Although α4-helix rigidity is a known feature of modern Trxs, it remains unclear whether this trend continues or reverses during cold adaptation. In this study, we used *Sphingomonas* sp. PAMC 26621 Trx (SpTrx) as a model to examine α4-helix adaptations in a cold environment. SpTrx shares 64% sequence identity with EcTrx and 42% identity with LBCA Trx, based on Clustal Omega alignment.^28^ While the two α2−α4 salt bridges are conserved across ancestral, thermophilic, mesophilic, and psychrophilic Trxs, the α4−β5 interaction diverges: LBCA Trx has an Asp87–Arg101 salt bridge, thermophilic Trxs retain a salt bridge at this position (Glu87–Lys101 in TtTrx), EcTrx features an Ala88–Phe102 hydrophobic pair, and psychrophilic SpTrx has an Ala87–Trp101 hydrophobic pair (Figure 1B–E). These variations reflect distinct strategies for stabilizing the α4 helix across thermal environments. To test whether Trp101 enhances α4-helix rigidity in cold-adapted Trxs, we introduced mutations in SpTrx targeting salt bridges (E43A, E47A, E43A/E47A), hydrophobic packing (W101A, W101F), and both (E47A/W101F). E47A was selected over E43A for the double mutant because it disrupts salt bridges with both Lys95 and Lys99. Corresponding mutations were introduced in EcTrx: E44A, E48A, and E44A/E48A for salt bridges; F102A and F102W for hydrophobic interactions; and E48A/F102W for both. We assessed stability using thermal denaturation, chemical unfolding, fluorescence, and CD spectroscopy, and measured enzyme activity via insulin reduction and DTNB kinetics. Our findings show that α4−β5 hydrophobic packing is the dominant stabilizing force in SpTrx, offering a distinct mechanism to maintain activity in cold environments.

## 2 Materials and Methods

### Materials

*Sphingomonas* sp. PAMC 26621 was obtained from the Korea Polar Research Institute (Incheon, South Korea).^25^ The *ectrx* gene in the pET32 vector was a gift from T. J. Kappock (Addgene plasmids #71699), and the pET28 vector was purchased from Novagen (Madison, WI, USA). nPfu-Forte polymerase and the restriction enzyme DpnI were provided by Enzynomics (Daejeon, South Korea). Chromatography columns (HisTrap, Capto Q, Superdex 200 XK16) were purchased from (Cytiva, Marlborough, MA, USA). Bis-ANS (4,4′-dianilino-1,1′-binaphthyl-5,5′-disulfonic acid, dipotassium salt) and DTNB (5,5′-dithio-bis­(2-nitrobenzoic acid)) were purchased from Thermo Fisher Scientific (Waltham, MA, USA). Other chemicals were obtained from Sigma (St. Louis, MO, USA) and Tokyo Chemical Industry (Tokyo, Japan).

### Site-Directed Mutagenesis

Site-directed mutagenesis was performed using nPfu-Forte polymerase to introduce mutations into the *sptrx* and *ectrx* genes cloned in the pET28 and pET32 vectors, respectively. PCR primers used for mutagenesis are listed in Table S1. Each 20 μL PCR reaction contained 9 μL PCR-grade water, 5 μL dNTPs (2.0 mM), 2 μL 10× Pfu buffer, 2 μL plasmid template, 1 μL primer, and 1 μL nPfu-Forte polymerase. Thermal cycling conditions were: initial denaturation at 95 °C for 2 min; 22 cycles of 95 °C for 30 s, 55–60 °C for 30 s, and 72 °C for 6 min; followed by a final extension at 72 °C for 10 min. PCR products were digested with DpnI at 37 °C for 1 h to eliminate the parental plasmids. The resulting mutant plasmids were transformed into *E. coli* BL21­(DE3) by heat-shock, recovered in antibiotic-free LB medium at 37 °C for 1 h with shaking, and plated on LB agar containing kanamycin (100 μg·mL^–1^) for SpTrx or ampicillin (100 μg·mL^–1^) for EcTrx. Mutations were confirmed by Sanger sequencing.

### Structural Analysis and Multiple Sequence Alignment

Structural models of SpTrx wild-type (WT) and mutant forms of SpTrx and EcTrx were generated using AlphaFold 3, yielding high-confidence predictions with pLDDT (>90) scores.^26^ The experimentally determined structures of EcTrx and LBCA Trx were examined using ChimeraX (version 1.9).^27^ Multiple sequence alignment of Trx orthologs was carried out with Clustal Omega,^28^ while residue intermolecular interactions were assessed via the Residue Interaction Network Generator,^29^ and the CaPTURE program for cation-π interaction.^30^

### Protein Expression and Purification

*E. coli* BL21­(DE3) cells harboring SpTrx or EcTrx constructs were cultured overnight in LB medium containing kanamycin (100 μg·mL^–1^ for SpTrx) or ampicillin (100 μg·mL^–1^ for EcTrx). The overnight culture was inoculated into fresh LB medium and grown at 37 °C for 2 h. Protein expression was induced with 1 mM isopropyl β-D-thiogalactoside (IPTG) when OD_600_ reached 0.6–0.8. After induction, cultures were incubated at 30 °C for 8 h. Cells were harvested by centrifugation at 4000*g* for 15 min at 4 °C and resuspended in buffer A (50 mM Tris·HCl, 50 mM NaCl, 5 mM imidazole, 0.1 mM EDTA, pH 8.0). The resuspended cells were sonicated on ice (2 cycles of 5 s on/5 s off, 50% amplitude). Lysates were centrifuged at 10,000*g* for 30 min at 4 °C, and the supernatants were applied to a HisTrap column. Proteins were eluted at approximately 250 mM imidazole. Eluted fractions were further purified using a Capto Q column with a NaCl gradient (50–1000 mM) in buffer B (50 mM Tris·HCl, 1000 mM NaCl, pH 8.0). The column was pre-equilibrated with buffer C (50 mM Tris·HCl, 50 mM NaCl, pH 8.0). Target protein fractions were then loaded onto a Superdex 200 column, and the final eluates were pooled in buffer D (100 mM Tris·HCl, pH 8.0) supplemented with 5% glycerol and stored at −80 °C until use.

### Protein Thermal Stability Assay

Thermal stability was assessed using SYPRO Orange dye on a StepOnePlus Real-Time PCR system (Applied Biosystems, Thermo Fisher Scientific, Waltham, MA, USA). Protein samples (25 μM) were prepared in phosphate buffer (100 mM sodium phosphate, 100 mM NaCl, pH 7.2) containing 3× SYPRO Orange (from a 5000× stock), in a final volume of 20 μL. Samples were heated from 25 to 99 °C at a ramp rate of 1% per sec in continuous ramp mode. The melting temperature (*T*_m_), defined as the midpoint of thermal denaturation, was determined using Protein Thermal Shift v1.4 software (Applied Biosystems).

### Fluorescence Spectroscopy

Protein stability and structural changes were analyzed using intrinsic fluorescence, guanidinium chloride (GdmCl)-induced unfolding, acrylamide-induced quenching, and bis-ANS fluorescence assays. All measurements were performed at 25 °C using a Scinco FS-2 fluorescence spectrometer (Seoul, South Korea). For intrinsic fluorescence measurements, excitation was set at 280 nm, and emission spectra were recorded from 300 to 400 nm.

### GdmCl-Induced Protein Unfolding

Intrinsic fluorescence was used to assess protein unfolding in response to GdmCl. Proteins (16.7 μM) were incubated in denaturation buffer (50 mM potassium phosphate, pH 7.5) containing 0–6.0 M GdmCl (in 0.5 M increments) for 30 min at 25 °C prior to fluorescence measurements. The equilibrium constant (*K*_eq_) was used to calculate the unfolding free energy (Δ*G*_H_2_O_^0’^) using the equation:

$$\Delta G_{H_{2}O}^{0\prime }=-RT\ln(K_{\mathrm{eq}})$$ I

where *R* is the gas constant (8.314 J·mol^–1^·K^–1^) and *T* is the temperature in Kelvin.^31^ All unfolding transitions were fitted to a two-state model to determine *m* values and baselines, assuming a linear relationship between Δ*G* and GdmCl concentration.

### Acrylamide-Induced Fluorescence Quenching

Acrylamide quenching was used to assess protein dynamics. Proteins (13.3 μM) were incubated at 25 °C in buffer B with acrylamide at concentrations ranging from 0 to 0.5 M (in 0.1 M increments) for 2 min before fluorescence measurement. Stern–Volmer plots were generated by plotting *F*_0_/*F* against acrylamide concentration [*Q*], where *F*_0_ and *F* represent fluorescence intensities in the absence and presence of acrylamide, respectively. The quenching behavior was analyzed using the modified Stern–Volmer equation:

$$F_{0}/F=1+K_{\mathrm{SV}}[Q]$$ II

where *K*_SV_ is the Stern–Volmer constant, calculated from the slope of the linear region of the plot.^32^

### Bis-ANS Fluorescence for Tertiary Structure Analysis

Changes in protein tertiary structure were assessed using bis-ANS fluorescence. Proteins (0.17 μM) were mixed with 100 μM bis-ANS in 50 mM phosphate buffer (pH 7.5) and incubated for 30 min at 25 °C. Fluorescence spectra were recorded using a Scinco FS-2 fluorescence spectrometer with excitation at 385 nm and emission from 400 to 700 nm.

### Far-UV Circular Dichroism (CD) Spectroscopy

Far-UV CD spectra (190–260 nm) were recorded at 25 °C using a JASCO J-1500 spectropolarimeter (Tokyo, Japan) at the Korea Basic Science Institute (Ochang, South Korea). Protein samples (58.3 μM, 200 μL) were prepared in CD buffer (50 mM potassium phosphate, pH 7.5) and incubated at 4 °C for 1 h prior to measurement. Spectra were acquired using a 1 mm path length cuvette with a 1.0 nm bandwidth, 0.1 nm data pitch, and a scanning speed of 100 nm·min^–1^. Each spectrum represents the average of three consecutive scans with a 1-s integration time. Data are presented as residual ellipticity (mdeg) versus wavelength and analyzed using GraphPad Prism (version 10). Secondary structure components (α-helix, β-sheet, turn, and other) were quantified using the BeStSel server.^33^

### Insulin Reduction Assay

The reaction mixture (800 μL) contained insulin (0.15 mM), SpTrx proteins (13.5 μM), and buffer (100 mM sodium phosphate, pH 7.0, 2 mM EDTA, 0.33 mM dithiothreitol [DTT]). Reactions were incubated at 25 °C, and absorbance at 650 nm was recorded every 5 min for 50 min using a Shimadzu UV-1800 spectrophotometer (Kyoto, Japan) to monitor precipitation of the insulin β-chain, as described in the original assay and adapted in subsequent studies.^34^ Precipitation onset was defined as a ≥0.02 increase in absorbance over baseline. The precipitation rate (Δ*A*_650_/min) was calculated during the linear phase (absorbance 0–1.0).

### Kinetics of DTNB Reduction

The kinetic parameters for 5,5′-dithiobis­(2-nitrobenzoic acid) (DTNB) reduction by SpTrx WT and its mutants were determined under steady-state conditions at their apparent optimal temperatures. SpTrx was prereduced with 5 mM DTT and used at a final concentration of 0.4 μM in reaction buffer containing 50 mM 3-(N-morpholino)­propanesulfonic acid (MOPS) (pH 7.0) and 1 mM EDTA. Reactions were initiated by adding DTNB (10–150 μM), and the formation of 2-nitro-5-thiobenzoate (TNB) was monitored spectrophotometrically at 412 nm at the respective optimal temperatures. Absorbance changes were quantified using an extinction coefficient of 28,300 M^–1^·cm^–1^, accounting for the production of two TNB molecules per reduced DTNB. The Michaelis constant (*K*_m_) and turnover number (*k*_cat_) were calculated from Lineweaver–Burk double-reciprocal plots, and kinetic data were fitted using GraphPad Prism software (version 10).

## 3 Results

### Protein Expression and Purification

Recombinant WT and mutant SpTrx and EcTrx proteins were expressed in *E. coli* BL21­(DE3) as soluble proteins and purified using HisTrap nickel-affinity and Capto Q anion-exchange chromatography. SDS-PAGE confirmed a single ∼12 kDa band for both WT and mutant proteins (Figure 2).

**Figure 2:**
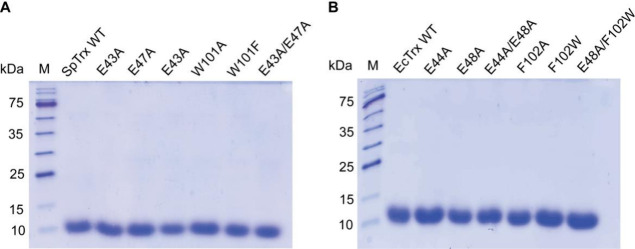
SDS-PAGE analysis of WT and mutant SpTrx and EcTrx proteins: (A) SpTrx and (B) EcTrx. M, molecular weight marker.

### Thermal Stability Analysis

The thermal stability of SpTrx WT and its mutants was assessed using SYPRO Orange-based thermal shift assays (Table 1, Figure S1). All SpTrx mutants showed reduced *T*_m_ values compared to WT (74.3 °C). Mutations at Trp101 caused the greatest destabilization, with W101A and W101F exhibiting *T*_m_ values of 57.4 and 61.8 °C, respectively, indicating the crucial role of Trp101 in stabilizing the α4 helix. In contrast, salt bridge mutants E43A (69.4 °C), E47A (66.9 °C), and E43A/E47A (65.1 °C) showed more moderate reductions, suggesting a secondary role for electrostatic interactions. Notably, E47A—which disrupts salt bridges between Glu47 and both Lys95 and Lys99—showed a lower *T*_m_ than E43A, which disrupts only the Glu43–Lys95 interaction. The E47A/W101F double mutant, which could only be analyzed for *T*_m_ due to poor expression, exhibited the lowest stability (51.7 °C), consistent with cooperative destabilization when both interaction types are disrupted.

**Table 1:** *T*_m_ Values of SpTrx, EcTrx WT, and Mutants^a^

SpTrx	*T*_m_ (°C)	EcTrx	*T*_m_ (°C)
WT	74.3 ± 0.1	WT	80.4 ± 0.1
E43A	69.4 ± 0.1	E44A	73.8 ± 0.2
E47A	66.9 ± 0.3	E48A	66.7 ± 0.2
E43A/E47A	65.1 ± 0.1	E44A/E48A	63.4 ± 0.1
W101A	57.4 ± 0.2	F102A	59.4 ± 0.1
W101F	61.8 ± 0.2	F102W	84.0 ± 0.2
E47A/W101F	51.7 ± 0.2	E48A/F102W	74.6 ± 0.1

a The data presented are the mean ± SD of three biological replicates.

### Conformational Stability Analysis

The conformational stability of SpTrx WT and its mutants was assessed by monitoring GdmCl-induced unfolding using fluorescence spectroscopy at 25 °C (Table S2, Figures 3, and S2). As SpTrx contains four Trp residues with only one Trp in α4-β5 interface and no Tyr in the structure, fluorescence changes reflect the local environments of Trp residues. All mutants showed reduced stability compared to WT, as indicated by lower denaturation midpoint ([D]_1/2_) values: W101A (1.7 M) < W101F (1.9 M) < E43A/E47A (2.1 M) < E47A (2.3 M) < E43A (2.5 M) < WT (2.7 M). Unfolding free energies (Δ*G*_H_2_O_^0’^) showed a similar decreasing trend. Substitutions at Trp101 caused the greatest destabilization, underscoring its crucial role in maintaining structural integrity through α4−β5 hydrophobic packing. W101A was particularly destabilizing, suggesting that smaller residues cannot substitute for the bulky indole side chain. In contrast, α2−α4 salt bridge mutants showed moderate destabilization, with additive effects observed in the double mutant. These results highlight the dominant stabilizing role of Trp101, with α2−α4 salt bridges providing secondary support.

**Figure 3:**
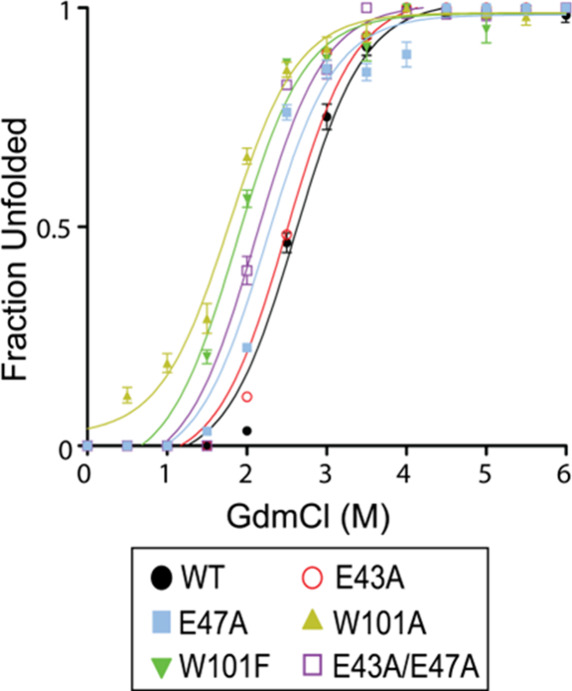
GdmCl-induced unfolding of SpTrx WT and mutants. Fluorescence spectra were measured after 30 min incubation at 25 °C with 0–6 M GdmCl (excitation 280 nm). Data represent the mean ± SD of three biological replicates.

### Conformational Flexibility Analysis

The conformational flexibility of SpTrx WT and its mutants was evaluated using acrylamide-induced fluorescence quenching (Table S3, Figure 4). Free Trp served as a baseline control to distinguish intrinsic indole fluorescence from protein fluorescence. All mutants exhibited lower inverse Stern–Volmer constants (*K*_SV_^–1^) than WT (0.58 M), indicating increased overall flexibility: W101A (0.11 M) < W101F (0.19 M) < E43A/E47A (0.27 M) < E47A (0.38 M) < E43A (0.40 M). The markedly lower values for W101A and W101F reflect increased flexibility due to the loss of Trp101, a rigid fluorescence source buried in a hydrophobic environment involving Ala86 and Ala87. In contrast, the salt bridge mutants (E43A, E47A, E43A/E47A) showed intermediate increases in flexibility, with additive effects in the double mutant. These results highlight Trp101 as the dominant contributor to conformational rigidity, while α2−α4 salt bridges provide a secondary, localized structural role.

**Figure 4:**
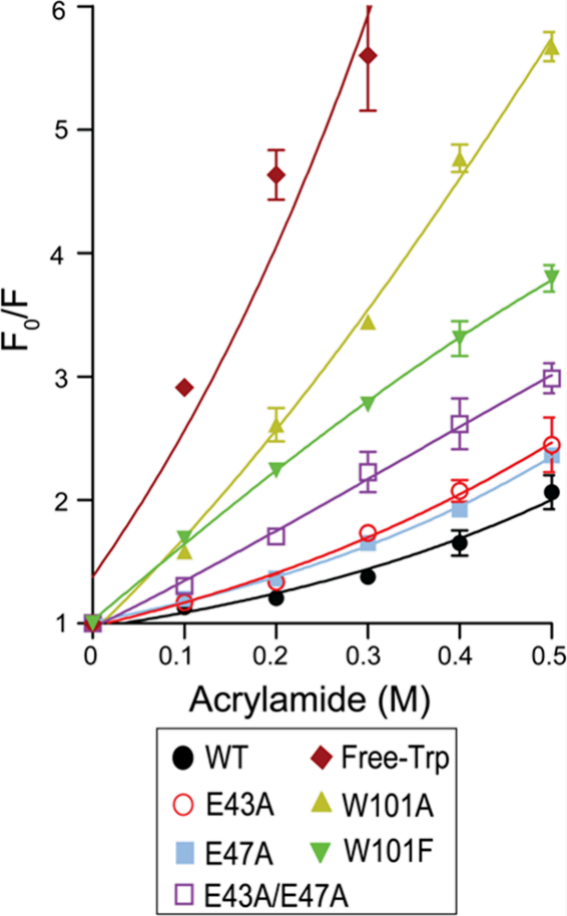
Stern–Volmer plot of SpTrx WT and mutants. Changes in fluorescence intensities were analyzed by plotting the ratio *F*_0_/*F* against acrylamide concentrations (0.1–0.5 M), where *F*_0_ is the fluorescence without acrylamide and F is the fluorescence at each concentration. The data presented are the mean ± SD of three biological replicates.

### Bis-ANS Fluorescence Analysis

Bis-ANS fluorescence, which detects changes in surface hydrophobicity, was used to assess the tertiary structure of SpTrx WT and two Trp101 mutants (W101A and W101F), as these substitutions directly affect the α4−β5 hydrophobic interface (Figure 5). WT exhibited the highest fluorescence intensity with a peak near 500 nm, consistent with tight hydrophobic packing at the α4−β5 interface. In contrast, both mutants showed reduced fluorescence, with W101A displaying the largest decrease, indicating disrupted packing. These results reinforce that replacing Trp101 with smaller residues weakens local hydrophobic packing and alters the tertiary structure.

**Figure 5:**
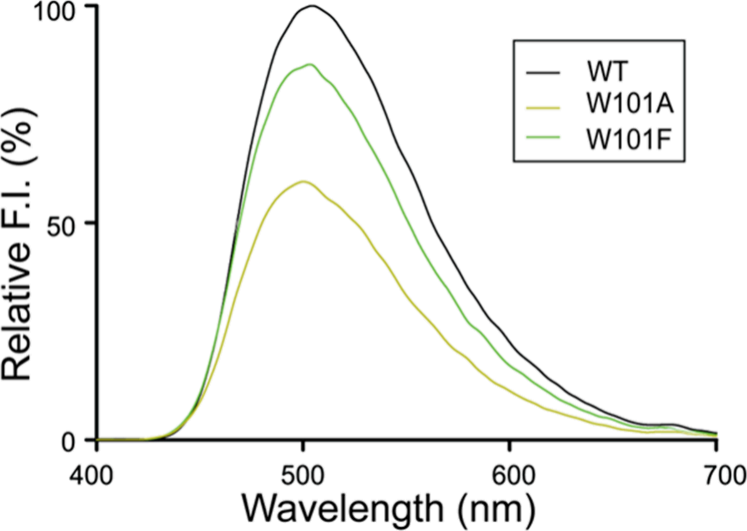
Bis-ANS fluorescence spectra of SpTrx WT and mutants (W101A and W101F). Proteins (0.3 μg) were mixed with 100 μM bis-ANS, and fluorescence spectra were recorded after a 30 min incubation at 25 °C (excitation at 385 nm). Data represent the mean ± SD of three biological replicates.

### Far-UV CD Analysis

The secondary structure of SpTrx and its mutants was assessed by far-UV CD spectroscopy (Table S4, Figure 6). The WT protein exhibited 24% α-helix content. Mutations that disrupted the α4−β5 hydrophobic interaction had the most pronounced effects: W101A showed the lowest α-helix content (11%) and a marked spectral shift at 222 nm, indicating extensive structural disruption beyond reduced stability. W101F, which retains hydrophobicity, showed a moderate decrease to 17%, suggesting partial loss of hydrophobic packing. In contrast, mutations affecting the α2−α4 salt bridges had milder effects, with E43A and E47A showing 27 and 20% α-helix content, respectively. The E43A/E47A double mutant dropped further to 15%, indicating additive destabilization. These results highlight the critical role of Trp101’s bulky aromatic side chain in maintaining secondary structure via the α4−β5 interface, while α2−α4 salt bridges provide secondary structural support.

**Figure 6:**
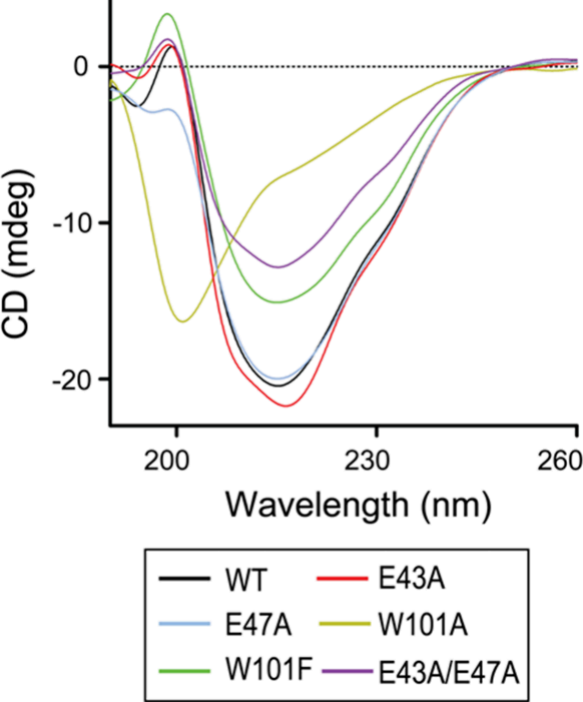
CD spectra of SpTrx WT and mutants. The spectra were measured at 25 °C, following a 1 h incubation of the proteins at 4 °C. Data are presented as the mean ± SD from three biological replicates.

### Catalytic Activity Analysis

The catalytic activity of SpTrx WT and its mutants was evaluated using insulin reduction assays at 25 °C and DTNB kinetic measurements at their apparent optimal temperatures (Table 2, Figure S3). Both assays showed consistent trends, with insulin reduction rates generally correlating with the DTNB-derived *k*_cat_ values. WT showed the highest activity, with an insulin reduction rate of 3.0 Δ*A*_650_ min^–1^ and a *k*_cat_ of 26.1 s^–1^. Mutations disrupting the α4−β5 hydrophobic interaction caused the most severe activity loss: W101A retained only 30% of insulin activity and 2% of *k*_cat_, while W101F retained 53 and 56%, respectively. In contrast, the single salt bridge mutants E43A and E47A retained ≥80% of insulin activity and 88% of *k*_cat_. The E43A/E47A double mutant showed further reductions—70% of insulin activity and 50% of *k*_cat_—despite lower thermal stability. These results underscore the crucial role of the α4 helix, particularly Trp101-mediated hydrophobic packing, in preserving catalytic function by stabilizing the active-site environment.

**Table 2:** Catalytic Activity for SpTrx WT and Mutants^a^

insulin reduction			DTNB kinetics		
	specific activity (Δ*A*_650_ min^–1^)	relative insulin activity (%)	*k*_cat_ (s^–1^)	*K*_m_ (μM)	relative *k*_cat_ (%)
WT	3.0 ± 0.1	100	26.1 ± 0.1	33.0 ± 0.1	100
E43A	2.8 ± 0.1	93	23.4 ± 0.1	38.3 ± 0.1	90
E47A	2.4 ± 0.1	80	23.0 ± 0.1	39.0 ± 0.1	88
W101A	0.9 ± 0.3	30	0.6 ± 0.1	59.9 ± 0.2	2
W101F	1.6 ± 0.1	53	14.6 ± 0.1	50.7 ± 0.6	56
E43A/E47A	2.1 ± 0.1	70	13.1 ± 0.2	44.0 ± 0.1	50

a Data presented are the mean ± SD of three biological replicates.

### Comparative Study with EcTrx

*T*_m_ values were measured for all EcTrx mutants (Table 1, Figure S1), with WT exhibiting a *T*_m_ of 80.4 °C. All mutants showed reduced thermal stability except F102W, which displayed an elevated *T*_m_ of 84.0 °C. While thermal stability was evaluated for all mutants, detailed structural analyses were conducted for WT, F102A, and F102W to examine the role of Phe102, which corresponds structurally to Trp101 in SpTrx. F102W showed greater resistance to GdmCl-induced unfolding ([D]_1/2_ = 3.9 M; Δ*G*_H_2_O_^0’^= 10.2 kcal·mol^–1^) compared to WT (3.3 M; 3.5 kcal·mol^–1^), along with reduced acrylamide quenching and increased bis-ANS fluorescence (Figure 7), indicating enhanced structural rigidity and tighter packing. In contrast, F102A showed decreased thermal and conformational stability, underscoring the importance of the bulky aromatic side chain at this position. These findings reinforce the critical role of the α4−β5 hydrophobic interaction in stabilizing the α4 helix of EcTrx.

**Figure 7:**
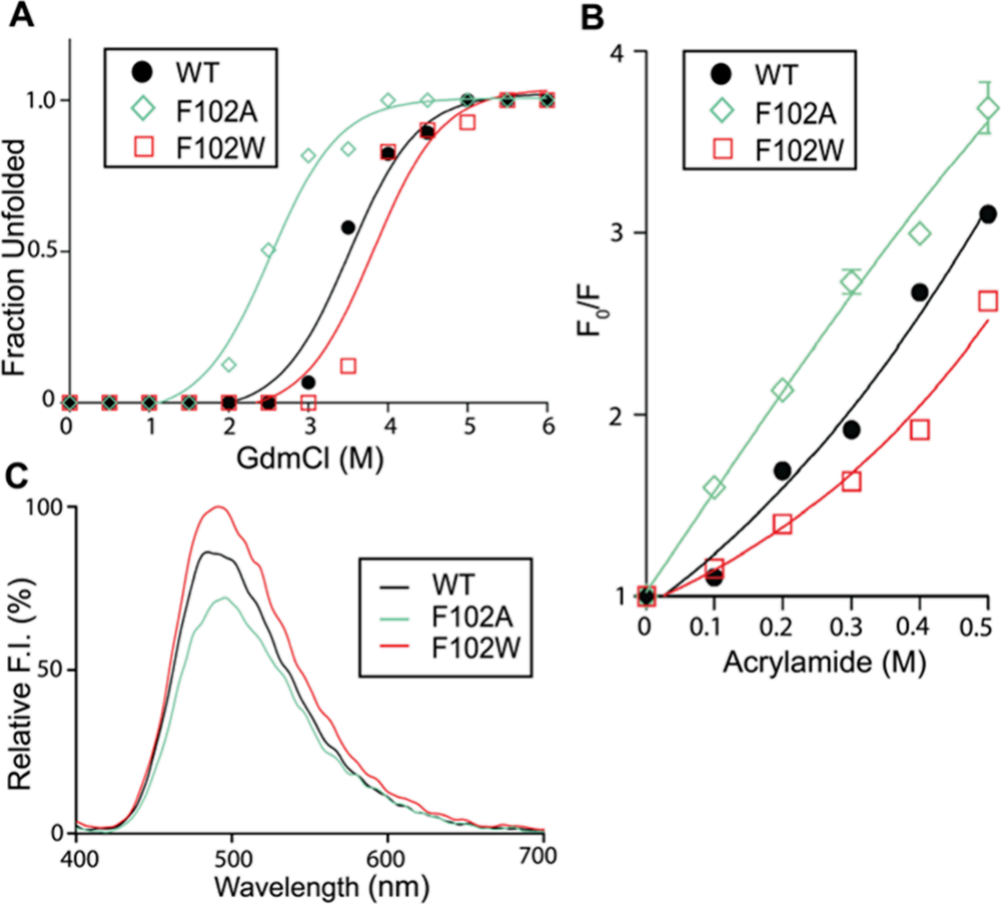
Structural analyses of EcTrx WT and mutants (F102A and F102W). (A) GdmCl-induced unfolding. Fluorescence spectra were recorded after 30 min incubation at 25 °C with GdmCl (0–6 M); excitation at 280 nm. (B) Acrylamide quenching. Stern–Volmer plots show *F*_0_/*F* as a function of acrylamide concentration (0.1–0.5 M). (C) Bis-ANS fluorescence. Proteins (0.3 μg) were incubated with 100 μM bis-ANS for 30 min at 25 °C; excitation at 385 nm. Data represent the mean ± SD of three biological replicates.

## 4 Discussion

Throughout Trx evolution from LBCA Trx to modern Trxs, the central β-sheet has remained structurally conserved.^12,16,35^ Hydrophobic interactions between the β2 and β4 strands are critical for the initial folding of Trx.^36,37^ In contrast, the α3 helix in EcTrx has become more flexible due to the loss of charge–dipole interactions involving ancestral Lys88, which has been replaced by Thr89.^16^ The C-terminal α4−β5 region has also undergone evolutionary changes: ancestral salt bridges (e.g., Asp87–Arg101) have been replaced by hydrophobic interactions, such as Ala88–Phe102 in EcTrx.^22^

Our results show that while cold-adapted Trxs are generally more flexible overall,^11,12,18^ the α4 helix of SpTrx becomes more rigid during cold adaptation. Trp101 plays a crucial role in stabilizing the α4−β5 interface, whereas α2−α4 salt bridges (Glu43–Lys95 and Glu47–Lys99) serve as secondary stabilizing elements. Ala substitutions at Glu43 or Glu47 moderately reduced thermal stability without significantly altering flexibility, secondary structure, or catalytic activity. In contrast, replacing Trp101 with Ala or Phe increased conformational flexibility and substantially reduced both stability and enzymatic function. The W101A mutant, in particular, exhibited the largest decrease in α-helix content, disrupted tertiary structure, and increased hydrophobic exposure—indicating broader structural defects beyond simple destabilization. These results suggest that strong hydrophobic packing via Trp101 reinforces α4-helix rigidity during cold adaptation and also compensates for weaker salt bridge contributions. Consistent with this, the E47A/W101F double mutant, which disrupts both α2−α4 and α4−β5 interactions, showed poor expression, likely due to impaired protein folding. These findings suggest that excessive flexibility—even in psychrophilic enzymes—can compromise structural integrity.^38^

In EcTrx, stability at the α4−β5 interface is primarily governed by the Ala88–Phe102 hydrophobic contact. The F102A substitution sharply reduced *T*_m_ by 21 °C—more than E44A (−6.6 °C) or E48A (−13.7 °C)—highlighting the dominant role of hydrophobic packing, with α2−α4 salt bridges playing a secondary, fine-tuning role.^39,40^ In contrast, the F102W substitution increased *T*_m_ by 3.6 °C, reflecting enhanced hydrophobic packing and possibly a stabilizing cation−π interaction with Lys90. To assess this, we built a model of the EcTrx F102W mutant based on the EcTrx crystal structure (PDB: 2TRX) and analyzed it using CaPTURE and RING. The analysis showed a cation−π interaction between Lys90 and Trp102, with a distance of approximately 4.5 Å, consistent with established criteria for a stabilizing interaction (Figure S4). By contrast, the thermophilic *Bacillus acidocaldarius* Trx retains a compact α2−α4−β5 salt bridge network, resembling the ancestral configuration.^41^ These findings are also consistent with previous work on a human–EcTrx chimera, where removing salt bridges reduced stability, while restoring even one (S44D or S44E) improved it.^42^

Cold-adapted enzymes are generally characterized by increased flexibility and reduced active-site stability, often due to structural features such as lower hydrophobic interactions, higher Gly-to-Pro ratios, increased Lys-to-Arg ratios, and extended loops—all of which promote efficient catalysis at low temperatures.^38,43,44^ However, SpTrx WT exhibited relatively high stability (*T*_m_ = 74.3 °C) and a *T*_opt_ of 60 °C (Table 1, Figure S3). All mutants—except E43A, which retained the same *T*_opt_—showed reduced *T*_opt_ of 40 °C, despite *T*_m_ values remaining above 40 °C. This indicates that the active site is more thermally sensitive than the overall structure,^43^ and highlights the role of C-terminal α4−β5 hydrophobic interactions in stabilizing the active site. This observation reinforces our previous findings on *Sphingomonas* sp. Grx3, where an α1−α3 salt bridge—structurally analogous to the α2−α4 salt bridges in Trx—helped stabilize the active site during cold adaptation.^45^ These results underscore the importance of α4-helix rigidity, maintained by Trp101, in stabilizing the active-site loop via the α4−β5 interface. In contrast, α2−α4 salt bridge mutations had relatively minor effects on activity.

## 5 Conclusions

Our results demonstrate that although Trxs have evolved increased overall flexibility to adapt to cold environments, they also retain—or reinforce—rigid structural features in key regions. In SpTrx, Trp101-mediated hydrophobic interactions at the α4−β5 interface are critical for local stabilization, supporting catalytic function despite overall flexibility. Additional studies, including molecular dynamics simulations or experimental probing of conformational dynamics, will be crucial to better understand the balance between flexibility and rigidity in cold-adapted Trxs.

## Supplementary Material



## Notes

List of primers used for site-directed mutagenesis and thermal denaturation profiles (SpTrx and EcTrx); stability parameters from GdmCl-induced unfolding, acrylamide quenching constants, secondary structure analysis by far-UV CD, two-state unfolding analysis, and optimal temperature profiles (SpTrx); and structural comparisons between EcTrx WT and the F102W mutant (PDF)

## Notes

SpTrx NCBI ID: WP_010164143.1. PsTrx NCBI ID: MDQ3140117.1. PtTrx NCBI ID: WP_015023308.1. PmTrx NCBI ID: WP_284204518.1. EcTrx PDB ID: 2TRX. PaTrx NCBI ID: KJJ13934.1. KaTrx NCBI ID: KLF75859.1. AaTrx PDB ID: 2I4A. TtTrx PDB ID: 2CVK. TaTrx NCBI ID: WP_053767495.1. LPBCA Trx PDB ID: 2YJ7. LBCA Trx PDB ID: 4BA7.

## Abbreviations

DTT: dithiothreitol
EDTA: ethylenediaminetetraacetic acid
Bis-ANS: 4,4′-dianilino-1,1′-binaphthyl-5,5′-disulfonic acid
DTNB: 5,5′-dithiobis­(2-nitrobenzoic acid
IPTG: isopropyl β-D-1-thiogalactopyranoside
NADPH: β-nicotinamide adenine dinucleotide phosphate
MOPS: 3-(N-morpholino)­propanesulfonic acid
