# Supplementary material for: Trp101-Mediated Cold Adaptation in Sphingomonas sp. Thioredoxin: Increased α4-Helix Rigidity with Preserved Overall Flexibility
Source: ACS Omega. 2025 Nov 4;10(45):54405–13. doi: 10.1021/acsomega.5c07089 (PMC12631696; doi:10.1021/acsomega.5c07089)
Supplement: Supplementary file 1 [file ao5c07089_si_001.pdf]

Supporting information for:

Trp101-Mediated Cold Adaptation in *Sphingomonas* sp. Thioredoxin: Increased α4-Helix Rigidity with Preserved Overall Flexibility

Mohammed Shazaly A. Elhassan,[1] Hoa Nguyen,[1] ChangWoo Lee [1,*]

[1]Department of Biomedical Science and Center for Bio-Nanomaterials, Daegu University, Gyeongsan, South Korea

* To whom correspondence should be addressed: Email: leec@daegu.ac.kr

Table S1. List of primers for site-directed mutagenesis.

		Forward (5'→3')	Reverse (5'→3')
SpTrx	E43A	cctcgagGCAatctcg	ctccgagatTGCctcg
	E47A	gaatctcggagGCActgggc	ctcgcccagTGCctccg
	W101A	gttgaaggcgGCGctcg	ccctcgagCGCcgcc
	W101F	cggttgaaggcgTTTctcg	cgagAAAcgccttcaaccg
EcTrx	E44A	cgattctggatGCAatcgc	gtcagcgatTGCatccag
	E48A	cgctgacGCAtatcaggg	gccctgataTGCgtcagc
	F102A	cagttgaaagagGCCctcgac	gcgtcgagGGCctctttc
	F102W	cagttgaaagagTGGctcgac	gcgtcgagCCActctttc

Mutated nucleotides are shown in capital letters. The double mutants were generated as follows: SpTrx E43A/E47A by introducing E43A into the E47A template; SpTrx E47A/W101F by introducing W101F into the E47A template; EcTrx E44A/E48A by introducing E44A into the E48A template; and EcTrx E48A/F102W by introducing F102W into the E48A template.

Table S2. Stability parameters for WT and mutants.

		$[D]_{1/2}$ [a] (M)	m [b] (kcal mol^{-1} M^{-1})	$\Delta G^{0'}_{H_2O}$ [c] (kcal mol^{-1})
SpTrx	WT	2.7 ± 0.01	2.9± 0.2	7.7 ± 0.5
	E43A	2.5 ± 0.01	2.5 ± 0.1	6.3 ± 0.4
	E47A	2.3 ± 0.01	1.7 ± 0.1	3.9 ± 0.2
	E43A/E47A	2.1 ± 0.01	1.5 ± 0.1	3.1 ± 0.1
	W101A	1.7 ± 0.02	1.2 ± 0.1	2.0 ± 0.1
	W101F	1.9 ± 0.05	1.4 ± 0.2	2.7 ± 0.2
EcTrx	WT	3.3 ± 0.03	1.1 ± 0.1	3.5 ± 0.2
	F102A	2.5 ± 0.01	2.0 ± 0.1	5.2 ± 0.1
	F102W	3.9 ± 0.03	2.5 ± 0.2	10.2 ± 0.8

[a] GdmCl concentration at which the unfolding transition midpoint is observed.

[b] Proportionality is constant between free energy and guanidium chloride concentration.

[c] Free energy of unfolding extrapolated to zero denaturants. Data presented are the means of three measurements.

Table S3. Inverse Stern–Volmer quenching constant (K_{sv}^{-1}) for SpTrx WT and mutants.

	K_{sv}^{-1} (M)
WT	0.58 ± 0.01
E43A	0.40 ± 0.01
E47A	0.38 ± 0.01
E43A/E47A	0.27 ± 0.01
W101A	0.11 ± 0.01
W101F	0.19 ± 0.01

K_{sv}^{-1} is the acrylamide concentration at which 50% of the fluorescence intensity is quenched.

Table S4. Secondary structure analysis of SpGrx3 WT and mutants.

	α-Helix (%)	β-Strand (%)	Turn (%)	Other (%)
WT	24	37	7	32
E43A	27	29	7	37
E47A	20	22	8	50
E43A/E47A	15	15	11	59
W101A	11	18	18	53
W101F	17	16	9	58

Data are reported as percentages of α-helix, β-strand, turn, and other structural content determined from far UV-CD spectra. The E47A/W101F double mutant was excluded due to insufficient yield from low expression and poor solubility.

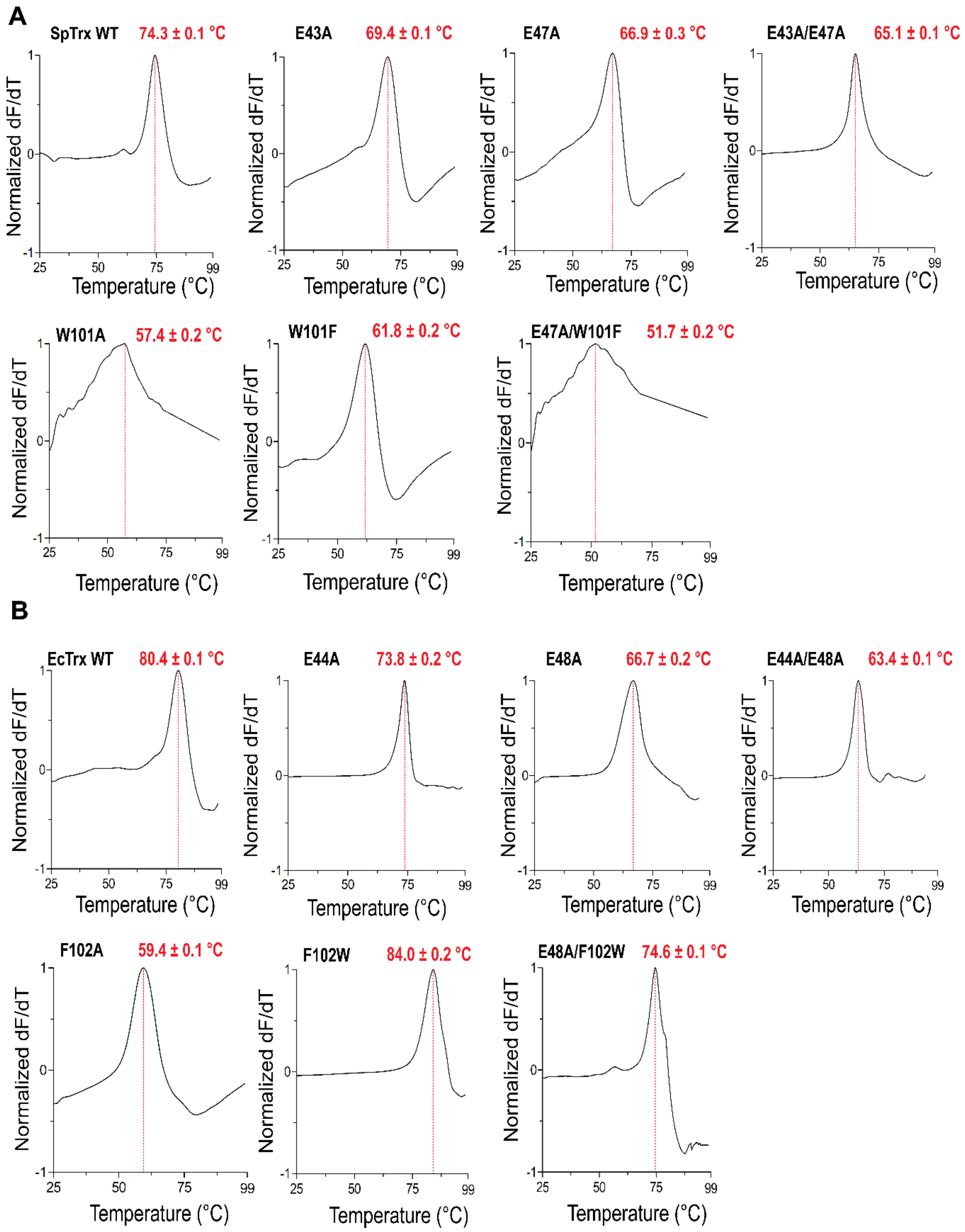

Figure S1. Melting temperatures of (A) SpTrx3 WT and mutants and (B) EcTrx3 WT and mutants. Proteins (0.3 mg/mL) were mixed with 3× SYPRO Orange dye (20 μL total volume), and thermal shift assays were performed using an Applied Biosystems StepOnePlus real-time PCR system. Data represent mean ± SD from three independent measurements.

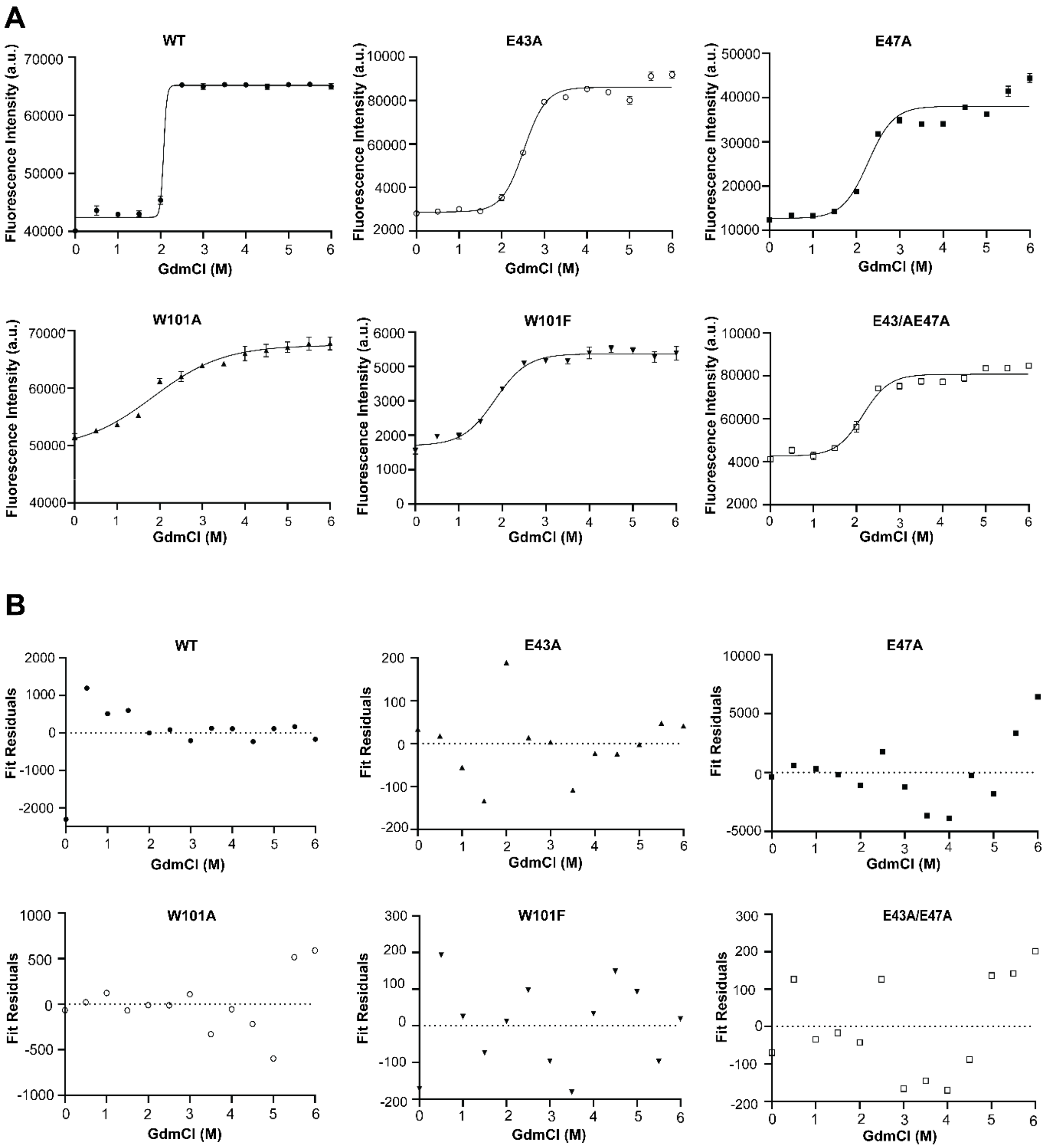

Figure S2. Two-state unfolding analysis of SpTrx WT and mutants. Denaturation was monitored by Trp fluorescence to assess the two-state unfolding model. (A) Denaturation curves. Experimental data (points, mean ± SD) with best-fit curves for WT and mutants. (B) Residual plots. Randomly distributed residuals support the validity of the two-state model.

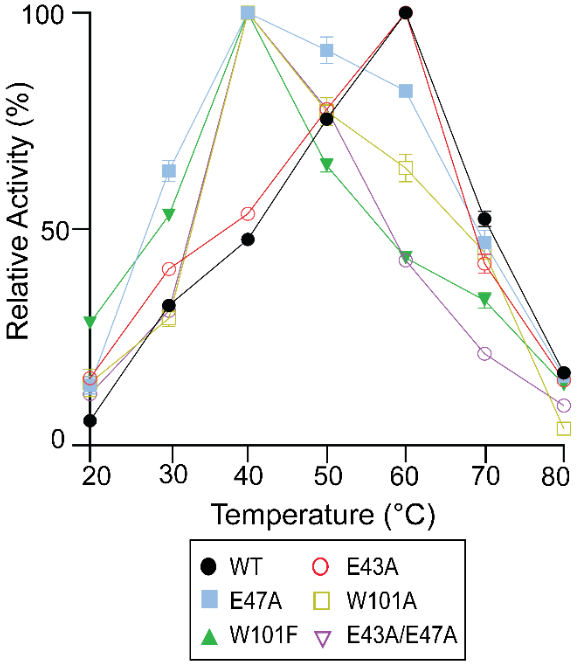

Figure S3. Apparent optimal temperatures of SpTrx WT and mutants. The activity at the optimal temperature for each protein was set to 100%. Data are presented as the mean ± S.D. of three biological replicates.

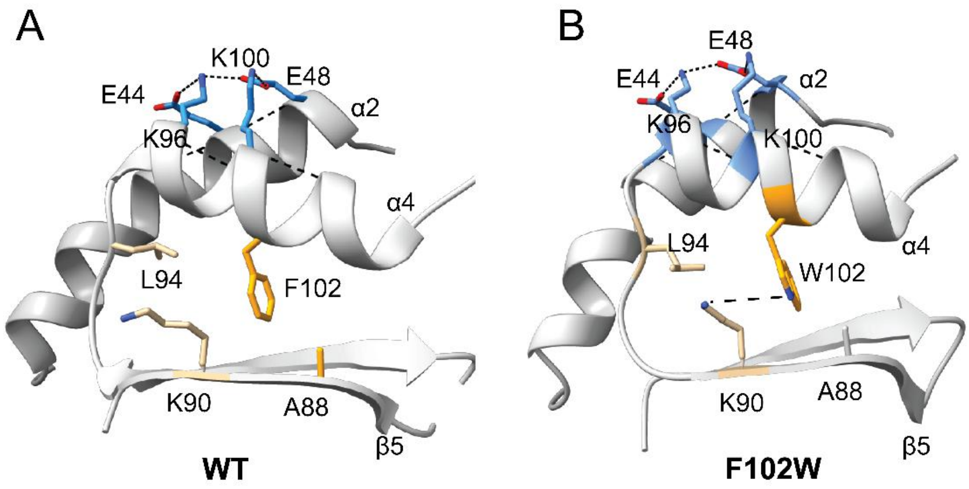

Figure S4. Comparison of α2–α4 and α4–β5 interactions in (A) EcTrx WT (PDB: 2TRX) and (B) the EcTrx F102W mutant. In F102W, a cation–π interaction between Lys90 and Trp102 is indicated. Structures were visualized and analyzed using ChimeraX (version 1.9).
